# Supplementary material for: Are carbon emissions trading and green financial instruments synergistic? -Comprehensive quantitative research based on content analysis
Source: PLoS One. 2024 Mar 7;19(3):e0298601. doi: 10.1371/journal.pone.0298601 (PMC10919601; doi:10.1371/journal.pone.0298601)
Supplement: S1 Data — (ZIP) [file pone.0298601.s001.zip › Data/地级市Z_F工作报告.zip!/┴╔─■/▒╛╧¬╩╨/└┤╫╘╣1⁄2╓┌║┼í╛┬φ┐╦╩2╛▌═°í┐_2014.docx]

各位代表：

现在，我代表本溪市人民政府向大会报告工作，请予审议，并请市政协委员和各位列席同志提出意见和建议。

2013年工作回顾

过去的一年，全市上下共同努力完成了各项年度任务。预计，全市生产总值完成1200亿元，同比增长10%；公共财政预算收入完成129.7亿元，按可比口径增长15%；固定资产投资870亿元，增长20%；规模以上工业增加值完成600亿元，增长11%；社会消费品零售总额实现295.2亿元，增长13%；实际利用外资5.5亿美元，增长15%；引进省外投资到位资金370亿元，增长25%；出口创汇26.5亿美元，增长10.2%；城镇居民人均可支配收入24825元，增长10.5%；农民人均纯收入11934元，增长10.5%。

一年来，市政府主要做了以下工作：

（一）深入开展项目年活动。以重大项目建设为抓手，采取市领导分工包扶、联合督查等措施，推动实施了511个重点项目。田桓铁路、千金岭隧道、观音阁输水工程等项目开工建设，本钢冷轧高强钢、思山岭铁矿、沈丹客专、丹阜高速石桥子互通立交改造等项目加快建设，本溪热电、大台沟铁矿、本桓高速等项目前期工作进展较快。同时，继续向上争取政策、资金等发展要素。我市被列为国家现代服务业综合试点城市，被纳入国务院批复的《全国老工业基地调整改造规划》。全年共争取上级资金66亿元，同比增长9%。经省政府批准用地102个批次，总面积600公顷。

（二）扎实推进钢都建设。进一步深化钢都建设内涵，实施“163”钢都发展战略。认真落实市政府与本钢集团签订的战略合作框架协议，解决了本钢集团“1+5”板块发展中遇到的13个难题。本钢集团深化转型升级，提升经济运营质量，在钢铁行业持续严峻形势下实现逆势健康发展，全年销售收入达到1100亿元，出口签约总量440万吨，连续四年在国内钢铁行业中实现出口总量第一，继续为我市经济平稳运行做出了决定性贡献。积极发展钢材深加工产业，北方曲轴、本溪工具等地方存量企业不断做优做强，银龙预应力、金桥焊材等全国行业领军的增量企业签约入驻并加快建设。钢材深加工产业实现工业增加值32亿元，增长38%。

（三）举全市之力推进中国药都建设。以7个重点产业和3个配套功能区建设为载体，继续实施“173”战略。坚持产业为重。围绕药都企业建设、投产达产中的相关问题，采取“点对点、一对一”服务企业措施，推进了华润三药、开普医疗等重点企业释放产能，推进了修正药业、九洲龙跃等15户企业投产运营，推进了恒康制药、药大药业等18户企业具备投产条件。通过“走出去、请进来”，引进上海绿谷、北京海凭、韩国大熊制药等20个医药产业项目。目前药都共集聚生物医药产业项目96个，全年实现销售收入240亿元，为建成产值千亿的中国药都奠定了坚实基础。坚持科技支撑。发挥8个省级公共技术服务平台的作用，为药都企业提供各类技术服务5000余项，储备在研新药项目293个，完成成果转化30项。基因科技水平全球领先的本溪华大基因研究院建成。药都被认定为国家首批10个、也是唯一的医药领域创新型产业集群试点，通过了国家创新药物孵化基地验收。采取“1+6”模式的科技、金融和产业结合工作全面启动，设立了总规模4.4亿元的“百川汇达”生物医药产业投资基金。坚持大学为要。辽宁医药化工职业技术学院、中国医科大学本溪校区一期投入使用；沈阳药科大学二期和辽宁卫生职业技术学院一期工程开工建设；辽宁中医药大学沈本医院、本溪校区分别投入使用和开工建设；辽宁科技学院首次获得国家“863”计划支持，省级生物医药与化学工程实验室投入使用。我市拥有高校数量跃居全省第三位，生物医药类高校正在形成集聚效应，高校具有的人才培养、科学研究、社会服务和文化传承等职能，将为我市的可持续发展提供有力的人才支持和智力支撑。坚持产城结合。实施道路、供电、河道治理等基础设施项目27项，会展中心、俊安金融广场等城市功能项目主体竣工。日月岛核心区用地完成30%的征迁任务。蓝远名城一期、御龙山语湾等6个房地产项目竣工。药都幼儿园开园招生。新开通63路和沈本城际巴士两条公交线路。目前，药都发展综合实力已进入国内同类园区的前5名。

（四）持续推进枫叶之都建设。大力实施“512”温泉旅游发展战略，希尔斯温泉小镇的温泉会馆等5个重点项目开工建设；汤沟国际养生度假中心、凤凰山及龙马塘温泉小镇等温泉旅游项目加快推进。实施“3个100”工程，本溪县特色旅游山庄建成运营9个、在建11个；桓仁县冰酒酒庄建成运营12个、在建29个；开发建设枫林谷等沟域旅游项目10个。本溪水洞通过国家5A级旅游景区评审。“十一”黄金周节日旅游市场持续火爆，冰雪旅游等冬季旅游不断升温，全年接待海内外游客增长13.2%，旅游总收入增长15%。同时，辽宁科技学院设立了旅游专业，为枫叶之都发展提供了人才支撑。

（五）统筹抓好工业、农业和服务业。工业经济平稳运行。本钢集团生铁、钢、热轧板等主要产品增幅均在10%以上。县区工业增加值增长20%，成为工业经济增长的重要力量。工业用电量、交通运输货运量等经济运行先行性指标均保持正增长。全年新增规模以上工业企业100户，完成工业固定资产投资400亿元。新建标准化厂房100万平方米，新入驻企业200户。9个工业产业集群实现销售收入840亿元。农业经济稳步发展。全市粮食产量达到29.8万吨，扎实推进了7000亩蔬菜基地的规划建设。市级以上农业产业化龙头企业达到126户。设施农业新建标准水稻育苗大棚167栋。以林下参为主的林地药材发展到130万亩。新建小型农田水利工程117项，新增节水灌溉面积1.05万亩。加强重大动物疫病防控工作，确保了畜产品安全。服务业加快发展。全年房地产新开工800万平方米、竣工600万平方米。推进了8个服务业集聚区的现代商业街、商业综合体等项目建设。完成19家农贸市场改造升级任务。新增152户限额以上服务业企业纳入统计范围。组织“夜经济”大型活动45次。新增两个省级物流示范园区。积极发展会展经济，成功举办了第五届药交会、第九届枫叶节等大型会展活动。全年服务业实现增加值410亿元，同比增长8.8%。

（六）不断壮大县区经济实力。两县均进入全省“一县一业”示范县行列，本溪县获批全国唯一的“林业产业示范县”，桓仁县建设了国家级冰酒检验检测中心。本溪县玉晶玻璃第三、四条生产线，桓仁县沈煤龙盟新型环保材料等重点产业集群重大项目建成投产，引进10亿元以上项目10个。两县房地产开工面积均达到100万平方米以上。小草线、迎水寺大桥和浑江大桥、民族广场等基础设施项目完成或主体竣工。两县县均生产总值和公共财政预算收入预计分别增长10.9%和13%。

城区工业园区和服务业集聚区竞相发展。桥北钢材深加工产业园已入驻规模以上企业58户，投达产企业50户。明山区新材料产业园山水水泥环保搬迁项目被列入国务院大气污染防治计划国省责任书。东风湖钢材深加工产业园总投资16亿元的恒通轧辊项目正式投产。总投资10亿元的山西继亨汽车配件项目落户南芬铸件产业园。4个城区服务业集聚区主营业务收入均突破50亿元。欧洲城、万鑫国际酒店等房地产和商业综合体项目扎实推进。城区平均生产总值和公共财政预算收入预计分别增长12%和19.1%。

（七）切实加大城市建设管理和生态环境保护力度。统筹主城改造和新城建设。新建、改造22条城市道路，地工路、环山路等18条道路竣工通车。北地跨线桥一期和太子河新城威龙路工程主体完工。维修整治100条街巷路、60条主次干路。栽植树木75万株，新增绿地26万平方米。有效治理了城区320条、500余公里道路两侧“五乱”、小招贴等违章行为。“创卫”工作5大攻坚战取得成效，10项必备条件已达标9项。沈本一体化有新进展，全域使用024区号获得国家工信部批准，顺利实现沈本工商登记管理一体化。启动了智慧城市建设工作。

继续实施“三大工程”。青山工程累计完成小开荒还林和退坡地还林7.2万亩，造林绿化6.6万亩。巩固碧水工程成果，完成了万吨以上污水处理厂水质验收工作。“创模”和蓝天工程加快推进，完成了66个重点污染治理项目，各项污染物排放总量控制在国家和省下达范围内，全年二级以上良好天数达到352天，空气环境质量名列全国68个重点城市第10位。编制并批准了国家生态市建设规划。启动村屯环境整治，完成两县47个整治示范区、122个项目村的农村环境连片整治示范项目验收工作。

（八）坚定不移推动改革开放。市政府实施了扁平化、一体化的管理体制创新，重点工作按照任务书、时间表、路线图推动。向城区下放权限24项。完成了事业单位分类工作。城商行增资扩股方案获省银监局批准。农信社改制稳步推进，桓仁农村商业银行在全省率先获批筹建。龙宝参茸公司上市申请获中国证监会受理。积极探索开放招商的体制机制创新，市政府班子成员带头扁平化、点面结合地推动开放，市直各部门和各县区也分层推动。全年共围绕生物医药、工业地产、服务业等专题，开展“走出去、请进来”招商活动49次，先后赴上海、香港、台湾、北京等地推介推进260多个项目。仲量联行、上药集团、首创集团、神州数码等国内外知名企业达成了在本溪投资合作意向。

（九）完成了十大类26件民生实事。一是实名制就业7.8万人；高校毕业生就业率达到95%以上；城镇登记失业率控制在4%以内。二是争取惠农资金1.02亿元；3.8万名农村低收入贫困人口脱贫；全市最低工资标准提高100元以上。三是多措并举，尤其是争取省以上专项资金29亿元，确保了企业离退休人员养老金按时足额发放；强力推进了社保扩面工作，符合条件的城乡居民参保比例达到90%以上；资助城乡低保及边缘对象8.4万人参加补充医疗保险；城市和农村月低保标准分别提高10.5%和12.2%。四是完成国有工矿棚户区改造等保障性安居工程9285套。五是开发建成10个微广场、21个停车场，新增停车位2961个；实施“暖房子”工程200万平方米，改造老旧管网108公里，295万平方米低温区得到改善，受益群众4.2万户；新建改造城市供水管网67.5公里；完成农村饮水安全工程63处。六是高新区入驻高校新增2所投入使用、2所开工建设；辽宁省实验学校本溪分校正式组建。七是全面落实了基本公共卫生均等化项目，全市电子健康档案规范化建档率达到82%；国家基本药物制度覆盖面扩大到全市309所村卫生室，实现人口全覆盖。八是建成高峪山、青年山、城中山3个体育主题公园；继续承办了CBA篮球联赛。九是在各县区建立各类托养照料服务机构27家。十是完成50个社区和500户贫困残疾人家庭无障碍设施改造；为100位农村单身贫困母亲援建住房。

同时，社会事业全面发展。完成33所幼儿园和20所城区义务教育相对薄弱学校改造建设任务；普通高中的国际交流、体音美等优质特色校建设成果惠及山城学生。市艺术宫改造全面启动；市人民文化宫改造成为群众文化活动阵地；辽砚、剪纸等文化产业实现新发展。引进高层次人才35人、研发团队12个。启动实施了国家级县级公立医院综合改革试点。圆满完成“十二运”本溪赛区赛事承办和安保工作。持续推进平安本溪建设，维护了全市治安大局稳定。我市连续三年获得全省安全生产工作先进市称号。信访稳定工作进一步加强。人口计生、外事、侨务、对台、民族、宗教、国防动员、人防、档案、史志、气象等工作也取得新进展。

（十）积极加强政府自身建设。市政府班子坚持月调度、周碰头和重点工作日报告制度，建立了每周一次的点对点服务企业（项目）工作机制，主动转变政府职能，提高行政效率。坚持依法行政，制定了市政府重大决策程序规定以及9项地方性法规草案和政府规章，办理行政复议案件261件。自觉接受人大依法监督、政协民主监督和社会监督，全年办理人大代表建议和政协提案955件，办复率100%、问题解决率72.1%。覆盖全市的四级公共行政服务体系基本形成，市公共行政服务中心受理办结审批事项230万件，办结率99.8%。行政监察和审计监督继续加强。“五大系统”全部进入运行阶段。各位代表，过去的一年，我们经受住了复杂多变外部环境的严峻考验，全市经济社会发展稳中有进。这是省委、省政府和市委正确领导的结果，是市人大、市政协监督支持的结果，是全市人民奋力拼搏的结果。在这里，我代表市政府，向全市各族人民、社会各界人士，向关心、支持和参与本溪发展建设的朋友们，表示衷心的感谢和崇高的敬意！

同时，我们也清醒地认识到：全市结构调整任务依然艰巨；财政收支矛盾依然突出；工业产业集群支撑力有待提升；服务业贡献率有待提升；一些关系群众切身利益的民生问题有待进一步解决；政府职能转变有待进一步深化等等。

2014年主要任务

2014年，我们要深入贯彻十八届三中全会精神，牢牢把握稳中求进工作总基调，进一步坚定信心，改革创新，稳中有为，加大力度推进新本溪建设。

新一年各项主要指标的计划安排是：全市生产总值增长10%；公共财政预算收入力争增长10%；固定资产投资增长20%；规模以上工业增加值增长10%；社会消费品零售总额增长13.5%；实际利用外资增长10%；引进省外投资到位资金增长15%；出口创汇增长10%；努力实现城乡居民收入与经济发展同步。

围绕实现上述目标，今年要着力抓好以下工作：

第一，以财税收入和项目建设为重点，全力打牢经济运行基础。针对全年财税收入目标建立倒逼机制，进一步完善各项主要指标的任务分解和推进落实机制，采取旬分析、月调度等措施推动工作，努力做到数量、质量同步提高。重点抓好主体税种和非税收入征管，加强财源建设，力争市本级公共财政预算收入实现同步增长。牢固树立过紧日子思想，以实施零基预算为切入点，优化政府财力配置，优先保障重点支出。持之以恒地开展项目年活动，努力推动451个亿元以上项目实现突破，83个新建、续建重大项目加快建设。重点推进辽宁中部环线高速本溪段、钢铁产业大道连接线、本溪热电等项目开工建设，沈丹客专、田桓铁路、观音阁输水工程等项目加快建设，丹阜高速石桥子互通立交改造、千金岭隧道工程等项目竣工，本桓高速等项目完成前期工作。同时，要继续向上争取政策、资金等要素；要继续谋划百个亿元以上重大项目；要继续解决重大项目建设进展问题。

第二，以形成“四个规模”为目标，全力发起药都建设总攻战。继续实施好“173”战略，创新体制机制，充分发挥国家级高新区的创新驱动载体作用，为我市实现稳中有为作出更大贡献。围绕形成经济规模和企业规模：一是进一步深化服务企业投产达产工作。继续主动服务企业，探索存量加增量“1+X”的发展效应。强力推进修正药业、未来生物等36户药业企业销售收入超亿元，推进恒康制药、药大药业等22户企业建成投产，引入华润医药、上海医药、四环药业等区域性总部和结算中心，全年药都投产企业达到75户、销售收入300亿元、实现税收15亿元。二是加大项目推进和储备力度。一方面以工业地产项目为载体，重点推进海凭国际医疗器械产业园、上海绿谷等项目加快开工建设，引进国内外生物医药、医疗器械等新项目50项。另一方面以张其寨地区为载体，打造健康服务产业集聚区，大力发展医疗服务、健康管理、健康养生等健康服务产业，继续推进东软熙康、韩国美容健康城等项目加快落地。三是继续发挥科技支撑和科技金融作用。推进“一心两园八平台”建设，支持沈阳药科大学建设国家级大学科技园，开发一批市场竞争力强、成果转化率高的创新药物及医疗器械新品种。按照“1+6”模式加强药都金融平台建设，设立科技融资担保和科技小贷公司，引进科技银行，再引进1家生物医药风险投资基金。围绕形成城市规模和人口规模：一是加速高新区特色房地产开发进程。全面推进日月岛片区开发建设，完善石桥子片区城市功能，启动开发张其寨片区，积极发展教育、温泉、养生、科技、总部等特色地产项目，力争高新区房地产新开工面积达到100万平方米，人口规模达到14万人。二是强力推进教育链项目建设。全力支持沈阳药科大学二期工程和辽宁中医药大学本溪校区主体完工，辽宁卫生职业技术学院一期工程投入使用，中国医科大学本溪校区全部投入使用并发展健康服务产业，支持省实验学校本溪校区实现招生办学。高新区将形成从学前教育到高等教育全链条的全省最优教育链。三是产城结合配置各种要素。完善沈本新城基础设施，启动实施路网、供电、供暖、绿化及广场、水系治理等基础设施项目33项。招商引进和建设商业综合体等商贸服务业项目，开工建设公安业务技术用房、移动公司综合楼等项目，办好第六届药交会，努力实现药都综合发展实力位次前移。

第三，以钢都建设为引领，推动工业产业集群加快升级。坚持“以钢为要、以钢带园、地企融合、共生共赢”的理念，谋划和实施好“163”钢都发展战略，全力打造科技含量高、规模效益好、资源消耗低、生态环境好的“中国本溪·绿色钢都”。重点建设中国精品板材和棒线材、桥北钢材深加工、中国人参铁加工、中国钢铁精品原材料、本溪湖冶金资源综合利用和辽宁（明山）新材料6个产业基地，培育钢铁产品物流配送、冶金科技与创新服务、中小企业融资服务3个中心。继续落实好地企合作协议，全力服务本钢集团，支持本钢集团开发一铁二电地区等重点项目建设。同时，继续深挖本钢等龙头企业的产业链上下游企业，开展专业招商、以商招商，不断提升钢材深加工产业规模和质量。平山区要继续发挥服务本钢集团和发展钢材深加工产业排头兵的作用。在做强传统优势产业的同时，加快玻璃制品、葡萄酒和包装印刷等产业发展，推进我市工业产业集群形成集聚效应，重点抓好工业地产的招商、建设，9个工业产业集群实现销售收入1050亿元。落实工业“五项工程”任务，滚动推进400个投资千万元以上重点工业项目，完成工业固定资产投资400亿元以上。围绕重点行业的规模以上重点企业进行全力支持服务，做到既做强存量、又吸引增量。继续实施《本溪市中小企业促进条例》，鼓励支持中小企业和非公经济做大做强。

第四，以枫叶之都建设为载体，推进旅游产业提档升级。进一步实施《“中国枫叶之都”建设行动纲要》，大力发展温泉旅游、乡村旅游、沟域旅游等特色旅游。落实温泉旅游“512”发展战略，发挥“中国温泉之城”品牌优势，通过温泉旅游项目的多点布局，形成具有我市特色的温泉、滑雪、枫叶等多种旅游业态。本溪县要在温泉旅游重点项目建设中取得突破性进展，希尔斯温泉小镇的温泉会馆等项目、汤沟国际养生度假中心香格里拉酒店项目均要主体完工。桓仁县青云寺国际温泉滑雪小镇项目一期运营。依托本溪生态资源特色，继续培育自驾游、徒步游、写生游、采摘游、蜜月游等旅游产品，形成集观光、体验、休闲、养生、度假于一体的复合型乡村、沟域旅游。进一步完善吃住行游娱购等旅游要素，建设20个特色旅游山庄、20个葡萄酒酒庄，100个沟域旅游项目完成招商任务。全年旅游总收入增长15%以上。

第五，以服务业重点产业为抓手，努力形成多元化服务业发展格局。抓住建设国家现代服务业综合试点城市契机，探索电子商务等新模式，大力发展房地产、物流、金融业等现代服务业，积极挖掘传统服务业潜力，抓好生产性服务业分离工作，培育发展“夜经济”，围绕“三高两点”目标加快推进服务业发展提速、比重提高、水平提升。

加快发展房地产业。抢抓新型城镇化建设机遇，完善基础设施配套，强化政策引导，以主城区、沈本新城、两县县城为房地产开发重点区域，以商业、旅游、健康、温泉、教育、工业地产等需求导向型地产模式为载体，解决胜利路三角地开发、平山甲楼等难点项目，促进全市房地产市场健康有序发展。全年房地产业的税收增长要高于全省平均水平，建筑业实现税收增长10%。

加快发展物流业。围绕打造沿海经济型城市，充分发挥物流行业协会主体作用，采取规划引导、资源整合、市场运作等方式，推动全市物流业向规模化、产业化方向发展。重点发展钢铁物流，努力打造东北最大的钢铁产品物流配送中心；发展医药物流，支持天士力物流做大做强，引进康美药业等知名医药物流企业，将药都打造成东北的医药物流中心；引进旅游小镇等项目，做强旅游集散市场；推进建设一个集农副产品、土特产、小包装食品批发交易于一体的大型农产品交易市场。

加快发展金融业。积极引进股份制银行，支持国有商业银行，发展地方金融机构。继续支持城商行加快发展，推动农信社完成改制任务，培育发展村镇银行、小额贷款、融资担保、保险证券等地方性金融机构和金融企业，全力推进龙宝参茸等企业上市，充分发挥人民银行本溪中心支行和本溪银监分局的服务监督作用，引导和鼓励金融机构支持实体经济。全市金融企业实现税收8亿元，增长40%以上。

加快发展城区经济。鼓励支持4个城区不断增强经济综合实力，对全市贡献要高于上年水平。4个城区要按照“七个一工程”要求，加快推动服务业集聚区向年主营业务收入超百亿元园区迈进，抓好五星级酒店、商业综合体、特色商业街的规划、招商和建设。平山区中央商贸商务集聚区建设要有实质性进展。明山区要做好“东进”和“北联”文章。溪湖区要在“原点变支点”和“南进北拓”上取得实质性进展。南芬区要在打造“特色发展先导区”方面实现新突破。

第六，以县域经济发展为依托，促进农业和农村经济较快增长。继续做好两县的“一县一业”提质增效工作，桓仁县以建设“世界冰酒之都”为目标，壮大酿酒葡萄产业示范基地；本溪县以建设中国药都优质原料生产基地为目标，积极发展中药材订单生产。两县工业产业集群要积极引进、培育投资超亿元的项目，两年内全力冲刺百亿元集群规模。城镇化要坚持县城和小城镇建设齐头并进，两县新开工房地产面积均要达到120万平方米。两县在全省44个县的综合实力排名要实现位次前移，对全市贡献要高于上年水平。同时，认真落实好省政府工作部署，扎实推进新型城镇化建设各项工作。

农业和农村经济要继续抓好“米袋子”、“菜篮子”建设，全年粮食作物播种面积达到70万亩以上；立足本地实际推进蔬菜基地建设，加快特色产业发展。培育推进4个超10亿元农业产业化重大项目。新发展各类林业产业基地18万亩，林业经济增长10%以上。农田水利建设要完成农村饮水安全工程15处、小型农田水利工程60项，新修、加固河道堤防60公里，建设河道生态工程1万亩。

第七，以十八届三中全会精神为指引，着力推进改革开放。做好省政府部署的22项具体改革任务准备衔接工作。结合本溪实际，当前抓好以下工作。一是深化行政审批制度改革。加强权力运行制度建设。推进投资体制改革和工商登记制度改革，进一步简政放权。建立健全政府向协会、中介组织等社会力量购买服务机制。二是深化政府机构改革。完成工商、质监管理体制调整，整合加强交通运输、卫生和计划生育、食品药品监管等领域和部门的职能和机构。同时加快事业单位分类改革。三是深入推进财政改革。继续扩大国库集中支付、政府采购、公务卡的实施范围和规模。建立融资偿债机制。推进项目绩效管理，建立健全绩效评价机制。加强国有资产监管，提高国有资产运营质量和效益。四是采取创新措施化解要素难题。通过积极争取新增建设用地指标、增减挂钩、低丘缓坡开发、盘活存量用地等方式，拓展用地空间，做到耕地占补平衡，实现国土部门对全市发展贡献高于上年水平。同时，加强土地储备工作领导，创新运行机制，提高贡献程度。制定出台有吸引力的人才政策，面向海内外引进一批高层次创新人才和急需紧缺的专业技术型人才。五是以招商引资体制机制创新推动对外开放。围绕主导产业和工业产业集群、服务业集聚区以及健康服务、物流经济、总部经济等方面，重点针对美国、欧洲和长三角、珠三角、京津唐等国家和地区，加大“走出去、请进来”力度，落实省政府对台经贸合作的“三个一”工程，推动全市联动招商，着力提升招商引资规模和对外开放水平。

第八，以建设生态宜居城市为方向，切实抓好城市建设管理和生态环境保护。坚持主城改造和新城开发同步进行。继续抓好“创卫”工作，开展城市“五乱”专项治理，努力达到国家卫生城市标准。加大居住区违法建筑管理整治力度，溪湖区要发挥示范作用。实施园林绿化提升和市容环境净化工程，城区栽植树木30万株，新建、改造绿地30万平方米。加大生态文明建设力度，推进“创模”和国家生态市创建工作，确保全市水环境功能区达到国家和省考核标准、空气环境质量达到国家新标准。继续实施三大生态工程。青山工程完成超坡地还林1.23万亩，植树造林3.55万亩；碧水工程继续巩固建设成果，确保污水处理厂稳定达标运行；蓝天工程实施56个大气治理项目，重点推进北台地区空气环境质量治理取得突破。桓仁县先行，按照“五美”要求深入推动全市农村环境整治工作，继续改善农村面貌。以市民卡、云计算数据中心等项目建设为载体，整合资源建设智慧城市，打造“本溪·智慧山水城”。

第九，以保障和改善民生为落脚点，积极构建和谐社会。今年，市政府要继续办好十大类民生工程。一是围绕业有所就，今年城镇实名制就业6.5万人，城镇登记失业率控制在4.2%以内。二是围绕完善社会保障体系，继续巩固社保扩面工作，争取专项资金确保企业离退休人员养老金按时足额发放，适时提高失业保险待遇。三是围绕住有所居，实施城市棚户区和国有工矿棚户区改造工程，建设安置房1万户以上。四是围绕畅通道路交通，新建滨河北路溪湖至姚家段等6条道路，续建北地跨线桥等4条道路；改造街巷路100条；综合整治人行道20万平方米。建设村内道路200公里。五是围绕方便群众生活，提高旧住宅小区物业管理覆盖面，明显改善居住生活环境和物业管理服务水平，明山区要发挥示范作用。大力推进旧住宅区整体改造，完成“暖房子”工程200万平方米。全面完成主城区30家重点农贸市场改造升级任务。六是围绕群众关注热点，依法解决居民房屋未确权问题，平山区要发挥示范作用。解决居民停车难问题，探索建设立体停车场，新增停车位1000个以上。七是围绕学有所教，谋划教育强市工作，优化教育布局，完成省教育强县（区）年度任务。八是围绕文化惠民，发挥市人民文化宫等文化阵地功能；完成市艺术宫改造主体工程；启动建设市文联本溪文艺家之家。九是围绕病有所医，深化医药卫生体制改革，继续提高社区卫生服务中心等基层医疗机构服务能力。十是围绕帮扶弱势群体，开展贫困残疾人“辅具助行圆梦行动”；扩大农村妇女“两癌”免费检查及救助项目覆盖范围。我们要全力做好这十大类民生实事，努力让人民群众年年看到新变化、年年得到新实惠！

同时，做好第三次经济普查工作。继续推动辽砚、剪纸、版画、演艺等文化产业发展。继续开展群众体育活动。继续抓好安全生产、信访稳定、食品安全等工作。继续全力推进平安本溪建设，继续积极探索“打防管控”一体化的管理模式。继续加强国防后备力量建设。继续做好人口计生、双拥、人防、外事、侨务、民族、宗教、档案、史志、气象等工作。

第十，以开展群众路线教育实践活动为契机，进一步加强政府自身建设。落实群众路线教育实践活动要求，继续坚持“执政为民、重在落实”的执政理念，全力打造人民满意政府。一是要以务实的作风抓落实。市政府班子将继续实施“四位一体”的工作推进机制，以亲力亲为的态度、务实高效的作风和一张蓝图绘到底的决心，心无旁骛地干、心底无私地干、心心相映地干、心甘情愿地干，加快推进全年各项工作。二是要以创新的思维抓落实。进一步解放思想，拓宽视野，坚持立足并跳出本溪，站在参与全国竞争、进入全球市场的角度去研究全市发展问题，通过政府引导、企业主体、市场配置、体制机制创新保障方式抓好经济运行，努力探索本溪的创业创新创优创意发展。三是要以自律的意识抓落实。自觉接受人大、政协和社会各界、新闻舆论监督，办理好人大代表建议和政协提案。坚持推进依法行政和法治政府建设。坚决落实市委改进工作作风、密切联系群众的36条实施意见，切实加强政府自身建设，加大行政监察和行政问责力度，打造诚实诚信、勤政廉政、公众认可、群众满意的服务型政府。

各位代表、同志们，让我们在省委、省政府和中共本溪市委的正确领导下，改革创新，稳中有为，不断凝聚促进发展的正能量，为“三都五城”建设取得新成效，为全市人民生活水平再有新提升，为全面建成小康社会实现新进展而努力奋斗！
